# Nutmeg Intoxication: A Case Report

**DOI:** 10.7759/cureus.46286

**Published:** 2023-09-30

**Authors:** Marc J Casale, Lisa-Qiao L MacDonald, Avir Mitra

**Affiliations:** 1 Anesthesiology, Icahn School of Medicine at Mount Sinai, New York City, USA; 2 Emergency Medicine, Icahn School of Medicine at Mount Sinai, New York City, USA

**Keywords:** drug overdose, hyperactive delirium, toxicology, nutmeg, intoxication

## Abstract

Nutmeg intoxication is an uncommon precipitant of hyperactive delirium with severe agitation (HDSA) with anticholinergic properties that require a high index of suspicion for diagnosis. We present a case of a young adult who presented to the emergency department (ED) with HDSA. The patient was intubated and transferred to the medical intensive care unit (MICU) due to increasing safety threats unresponsive to multimodal de-escalation. He ultimately self-extubated, reported excessive nutmeg ingestion, and was discharged home after a short period of observation. Improved detection and streamlined management pathways for nutmeg intoxication will minimize unnecessary invasive procedures and costs to the healthcare system.

## Introduction

Hyperactive delirium with severe agitation (HDSA) is a common chief complaint in the emergency department (ED). Etiologies range from life-threatening to benign, and thus, diagnosis and management can be resource-intensive. Poison Control Centers have reported an increased incidence of calls relating to nutmeg intoxication in recent years. In April 2020, a viral internet video inspired young people around the world to record themselves ingesting various amounts of powdered nutmeg dissolved in water [1]. Accurate detection of acute nutmeg intoxication in an ED patient can be exceedingly difficult and may overlap with an anticholinergic toxidrome.

The oldest mention of nutmeg poisoning dates back as far as 1576 [2]. In modern times, the first report dates to 1908 and concerns a case series of patients who present in delirious states with mydriatic pupils, burning abdominal pain, and occasional spasmodic jaw movements with recovery between four and 30 hours after ingestion. Other reports from the previous three decades report similar acute psychotic symptoms with associated anxiety or visual hallucinations and autonomic signs such as dry mouth, cutaneous flushing, tachycardia, abdominal pain, blurred vision, and urinary retention [3]. Nutmeg intoxication can also present almost identically to an anticholinergic toxidrome. Originally, it was believed the two could be distinguished through pupillary examination, with true anti-cholinergic syndromes presenting with mydriatic pupils and nutmeg intoxication presenting with miotic pupils. However, such reports have not been substantiated, and thus, this is not a reliable method of distinguishing the two [4].

## Case presentation

**Figure 1:**
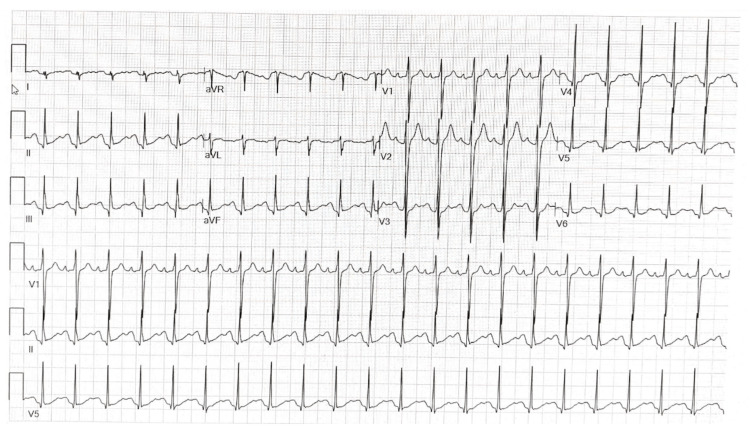
ECG taken at the time of admission demonstrating sinus tachycardia with normal intervals

Emergency medical services (EMS) presented a previously healthy 19-year-old male in police custody for aggression and suspected intoxication. EMS was activated after the patient bit another patron at a hotel bar. On arrival to the ED, the patient was calm and cooperative. A physical exam revealed tachycardia (129 bpm), fever (101℉), nystagmus, symmetrically dilated pupils with sluggish reactivity, dry skin, and a small forehead abrasion. There were no other signs of trauma, and the patient had no focal neurological deficits. The patient denied bowel or bladder incontinence. Electrocardiogram demonstrated sinus tachycardia with a narrow QRS interval, lowering concern for sodium channel blockage, and normal with QT interval, reassuring against organophosphate or tricyclic antidepressant (TCA) overdose (Figure 1). Initial labs were notable for elevated lactate (6 mmol/liter) (reference range: less than 1 mmol/liter). The complete blood count; basic metabolic panel; liver function tests; ethanol, salicylate, and acetaminophen serum levels; and a 10-point drug screen were unremarkable. Chest radiograph and head computed tomography (CT) scan were also unremarkable. Differential diagnosis included substances not included in standard drug screens (e.g., synthetic cannabinoids, mescaline, phenethylamine derivatives), polysubstance use, and central nervous system infection so antibiotics and lumbar puncture were pursued.

Shortly after ED arrival, the patient became both verbally and physically aggressive, including ripping monitors off the wall and throwing computers. He was unresponsive to verbal de-escalation so he was treated with intramuscular haloperidol (5 mg) and lorazepam (2 mg) three times. The patient’s agitation continued to worsen, posing a safety threat to others, despite repeated attempts at verbal de-escalation and multiple rounds of first-generation antipsychotic and benzodiazepine, so he was intubated to allow for continued management of HDSA.

The patient was admitted to the medical intensive care unit where he remained stable and self-extubated the following day. At this point, the patient was able to report that he took “a large amount” of nutmeg for its intoxicating effects, which was later corroborated by his family. Poison Control was contacted, and recommendations were 24-hour observation and supportive care. He remained stable throughout this time and was subsequently discharged at the end of this period.

## Discussion

We present a case of nutmeg intoxication associated with altered mental status, severe agitation, fever, and violence toward others requiring sedation and endotracheal intubation for both patient and staff safety. Intoxication with nutmeg was not recognized at the time of presentation. Reports of nutmeg intoxication in the literature are rare and rarer still are cases with agitation to the degree seen here. Accordingly, nutmeg misuse is not frequently part of the differential diagnosis when evaluating intoxicated patients in the ED.

There is a wide spectrum of clinical effects from nutmeg intoxication, and coingestions have been a confounding factor for studies that have aimed to characterize its toxidrome. Symptoms associated with nutmeg intoxication span a range of acuity and body systems: central nervous system (agitation, hallucinations) drowsiness, dizziness, blurred vision), gastrointestinal (nausea, vomiting, diarrhea), and cardiovascular (tachycardia, hypertension, hypotension). The majority of physical examination findings are either nonspecific or unreliable, with sinus tachycardia being the most consistent abnormal finding [2]. Other symptoms and related findings associated with an anticholinergic toxidrome include dry mouth, urinary retention, and blurred vision. Nutmeg intoxication remains a clinical diagnosis as diagnostic studies are not readily available in acute care settings.

Despite its long history of recreational use, the pharmacology of nutmeg intoxication remains poorly understood. The major chemical compounds of nutmeg are myristicin, elemicin, and safrole. Myristicin is partially metabolized to 3,4-methylenedioxy-5-methoxyamphetamine (MMDA) and elemicin is partially metabolized to 3,4,5-trimethoxyamphetamine (TMA), both amphetamine derivatives [5,6]. Accordingly, the psychoactive and autonomic components of nutmeg intoxication may be attributable to these metabolites; however, more recent studies failed to detect either of the amphetamine derivatives in the urine of patients with suspected nutmeg intoxication [7]. Another study also failed to identify any amphetamine derivatives in the urine of rats treated with myristicin, elemicin, or safrole using mass spectroscopy [8]. Myristicin is also known to be cytotoxic due to its partial metabolite myristicin-N-acetyl-cysteine, although it is still unclear if this compound contributes to the psychotropic and autonomic symptom profile of nutmeg [9]. Nutmeg extract, particularly trimyristin, has been shown to have anxiogenic effects that interfere with the activity of various drugs that act on serotonin receptors such as ondansetron and buspirone [10].

The minimum effective dose of nutmeg has not been defined. Several cases of intoxication have been reported after a 5 g nutmeg ingestion which corresponds to 1-2 mg of myristicin per kilogram body weight [11]. Another study demonstrated intoxication of all participants (n=22) after ingestion of 10 g of nutmeg powder which is equivalent to approximately two teaspoons of powdered nutmeg or three-fourths tablespoon of grated nutmeg [12].

Only two known cases of fatality associated with nutmeg have ever been reported. One was in an eight-year-old boy who consumed approximately 14 g of nutmeg and subsequently became comatose and expired 24 hours after ingestion [13]. The second involves a 55-year-old woman in which myristicin, one of the active ingredients in nutmeg, was found in the patient’s post-mortem serum [14].

Supportive care is the primary focus of treatment for nutmeg intoxication. Symptoms frequently resolve within 24-48 hours. Supportive care consists of cardiopulmonary monitoring and increased awareness of associated anxiety [3]. Benzodiazepines are particularly effective for anxiety and agitation associated with nutmeg intoxication [15]. There is also a possible benefit of activated charcoal in patients with intact airways [12].

## Conclusions

Despite low mortality and general complete recovery from nutmeg intoxication, it is essential for healthcare providers to be suspicious of potential nutmeg intoxication, especially when traditional toxicology screens are negative. Nutmeg is a cheaper alternative to consciousness-altering substances, and social media has contributed to its spread in popularity. As such, nutmeg intoxication should be considered in patients who present with an anticholinergic toxidrome associated with unknown substance ingestion.
